# Not EF skills but play with real toys prevents screen time tantrums in children

**DOI:** 10.3389/fpsyg.2024.1384424

**Published:** 2024-07-17

**Authors:** Margarita Gavrilova, Nikolay Veraksa

**Affiliations:** ^1^Federal Scientific Center of Psychological and Multidisciplinary Research, Moscow, Russia; ^2^Faculty of Psychology, Lomonosov Moscow State, University, Moscow, Russia

**Keywords:** child development, media use, screen time limits, screen time tantrums, play, executive functions, family activities

## Abstract

Limiting children’s screen time has become a new parenting challenge. Due to the high attractiveness of media and digital devices, many children experience painful transitions in screen time to other activities. Screen time tantrums is a new concept that describes children’s negative affect screen time limits. Knowing the factors that increase children’s negative reactions will be helpful in parents and educators practice to prevent screen time tantrums or enrich children’s nondigital activities, making them attractive alternatives to the media. Based on theoretical insights into the coping mechanisms of frustration and anger in preschool children, this study was aimed to explore the effects of executive functions skills, family activities, and children’s play behavior with real toys on screen time tantrums. Sample included 654 caregiver-child pairs (M children age = 70.3 months, SD = 4.02). Results confirmed the hypothesis that play behavior with real toys is a stronger preventor of screen time tantrum than EF skills. The findings suggest that supporting play activity in preschool children might help them to avoid strong negative affects due to screen time limitation. The results of this study may be also considered as a support for the theoretical assumptions that play can be considered as a universal way of coping with frustration and anger in childhood.

## Introduction

Studies indicate that excessive screen usage led to internalizing problems, with effects potentially lasting into school and adolescence (Staiano et al., 2018; Eirich et al., 2022). Still children actively use media (Lemish et al., 2018). Watching videos and playing digital games quickly become their favorite activities (Edwards, 2014; Danby et al., 2018; Veraksa and Chichinina, 2022). Since it is almost impossible to create a digital-free environment for children, it is important investigate the factors that interfere with attempts to limit children’s screen time. The current study examines whether play with real toys, executive functions skills, and family activities prevent screen time tantrums among children.

### Screen time tantrums and possible influences

According to the recent research, children miss the opportunity to develop useful skills and abilities in such activities as play, reading, art or communication (Bergmann et al., 2022; Nikolaeva et al., 2023). Because of lack of sufficient practice of these activities, children may not reach the level of mastery and concentration when they become enjoyable (Dolgikh et al., 2022). Moreover, other activities may even seem boring and not attractive to children and consequently, cause anger or frustration (Fitzpatrick et al., 2023). As a result, caregivers’ attempts to limit their children’s screen time will provoke intense emotional protest and tantrums (Coyne et al., 2021; Ishtiaq et al., 2021). Screen time tantrums is a new concept that describes children’s behavior in response to enforcement of screen time limits (Ishtiaq et al., 2021). Recent study reported that screen time limits caused tantrums in children in over 90% of families (Hiniker et al., 2016).

To date, there are only a few studies of screen time tantrums or painful screen time transitions (Hiniker et al., 2016; Coyne et al., 2021; Ishtiaq et al., 2021). Mainly, they survey the range of this problems in modern families, and the strategies applied by the caregivers to comfort their children. This is why current study cover potential family and individual factors that could influence the intensity of screen time tantrums in children. In regard to the family factors, it is essential to point out that the attempts to limit children’s screen time is only one of the problems related to the setting of boundaries in the parent–child relationship (Schroeder and Kelley, 2010). Family factors may include caregivers’ screen time and non-digital activities they share with their child (Hiniker et al., 2016).

Individual factors influencing children’s reaction to screen time transitions may include temperament, executive functions (EF) and emotional regulation skills (Gavrilova et al., 2023). Difficult temperaments significantly correlated with problematic reaction to screen time limits (Coyne et al., 2021). EFs that are defined as “cognitive processes that are required for the conscious, top-down control of action, thought, and emotions” (Müller and Kerns, 2015), are supposed to support the child’s coping with anger and frustration caused by painful screen time transitions (Veraksa and Veraksa, 2021).

Non-digital activities should significantly reduce potential tension caused by screen time transitions, and become an attractive alternative of a child’s pastime. Being intrinsically motivated and associated with pleasure and enjoyment, play holds a unique position among such activities (Veraksa and Sukhikh, 2020).

### Play as a possible screen time tantrum preventer

Play, such a natural and seemingly elementary activity, is of great importance to a child’s development. In cultural-historical approach and activity theory play is called the leading activity of preschool age because it promotes higher mental functions development (Vygotsky, 1997). Research confirms that through play self-regulation, academic and social skills are developed (Postholm and Vennebo, 2020; Bredikyte and Hakkarainen, 2023).

There are a number of play classifications, depending on what aspects of play are considered primarily: play actions, roles, plots, peer-to-peer interactions, etc. (Veraksa and Sukhikh, 2020). Some of these approaches may identify up to 16 types of play (Hughes, 2001). For all types of play, the common feature is that in it the child creates an imaginary situation and acts simultaneously in two dimensions: real and imaginary.

There are a number of potential reasons why play would be expected to reduce the problem of a screen time tantrum. Firstly, play is one of the most efficient ways to relieve frustration in early age. Vygotsky regarded it as an imaginary or an illusory form of fulfillment of impossible wishes (Vygotsky, 1997; Veraksa and Samuelsson, 2022). For a child, play is absolutely frustration-free, because everything is possible in it. This is why play is considered a self-motivated activity. In other words, the reason of its deployment is the possibility to fulfill one’s every wish. For example, a child who wants to be a doctor and heal people can achieve it in play. Despite that the child is a doctor only in this imaginary situation, the emotions he/she is experiencing are real, and the tension goes away for real as well (Veraksa and Sukhikh, 2021). In the psychoanalytic approach, the symbolic expression of unfulfilled wishes, conflicts, and negative emotions by the child is seen as a key characteristic (Wälder, 1933). Furthermore, the play psychotherapy is based on similar mechanisms of coping with frustration and similar negative emotions (McMahon, 2012).

Secondly, play guides the child in his/her exploring in the real world. Playing this or that role, the child discovers the meaning of human relationship and a whole range of adult activities (Fleer, 2013). Moreover, in the process of play the child practices interaction and cooperation with other children, which promotes emotional control development (Goldstein and Lerner, 2018) and his/her involvement in children’s communities. Play allows children to get more integrated into reality and obtain communication skills and building a closer relationship with the people around (Lenormand, 2018). Thus, play does not only help children to acquire a more detailed representation of the world, but can also enrich their emotional and communicative experience. According to Winnicott’s theory of play, this effect contributes to children’s wellbeing (Winnicott, 1991). Therefore, play can potentially be able to reduce the stress caused by screen time limits enforcement.

Thirdly, certain research indicate that play promotes metacognition, self-regulation (Robson, 2010; Whitebread and O'Sullivan, 2012), and self-development processes (King and Howard, 2016; Bredikyte and Hakkarainen, 2023) in children. This activity supports the development of these abilities because in it, children have to plan the plot and the characters’ actions independently, only relying on their own imagination. On the one hand, it is very advantageous because it encourages children to produce their own ideas (Fleer, 2022). It is also fundamentally different from digital entertainment, where children consume ready-made content. On the other hand, children learn how to implement their ideas. Being in the director’s position, a child creates an imaginary situation using the available means such as toys, objects, and environment, and performs play actions to bring his/her idea to reality (Veraksa et al., 2023). We could suggest that the skills of metacognition and self-regulation developed in this process can be successfully used by the child in the future for problem-solving in other life situations (Whitebread and O'Sullivan, 2012; Ryabkova and Sheina, 2023).

## Current study overview

This study is aimed at family and individual factors that can potentially relieve the manifestations of screen time tantrums in children. In particular, it evaluates the role of EF skills, play with real toys, and the frequency of such family activities (reading, singing, and drawing). By integrating the factors, which were mentioned earlier and might be potentially significant for screen time tantrums, this study should provide insight into the following research questions: Q1. Do well-developed EF prevent screen time tantrums? Q2. Does well-developed play with real toys prevent screen time tantrums? Q3. Does the frequency of family activities prevent screen time tantrums? This study also includes an exploratory analyses of play tendencies in children.

## Method

### Participants

The sample consisted of 833 5-year-old children and their caregivers. Only the participants (a) whose caregivers reported that their children used digital devices (see Measures) given our focus on screen time tantrums; (b) were born full-term with a weight 2,500 grams or above, in order to provide sample homogeneity (this factor can potentially affect the functioning of nervous system) were included in this study. One hundred and seventy-nine participants were excluded either because children did not use digital devices at all (*n* = 161), or their weight at birth was below 2,500 grams (*n* = 18). In total, 654 caregiver-child pairs participated in the study (M age = 70.3 months, SD = 4.02).

### Procedure

Kindergarten teachers handed out hard copies of questionnaires and detailed instructions. Each questionnaire had an envelope to be put in and sealed when it’s filled in. All the caregivers were assured of confidentiality and informed that the participation in the study was voluntary, and could be terminated at any moment. After the filled-in questionnaires were collected, the second stage of the study began that included individual assessment of the children’s EF skills (cognitive flexibility, working memory and inhibitory control). The study and consent procedures were reviewed and approved by Ethics Committee of Faculty of Psychology at Lomonosov Moscow State University (the approval no: 2022/21).

### Measures

#### EF skills

Cognitive flexibility was assessed using *The Dimensional Change Card Sort* measure (Zelazo, 2006). The test includes three trials, in each of which the child is asked to sort bivalent test cards according to one of three criteria (color, shape, and both at the same time that requires switching). The total score varies from 0 to 24.

Working memory was assessed using *Memory for Design* subtest of NEPSY-II battery (Korkman et al., 2007). This tool is designed to measure visual working memory by two parameters: spatial and content-based memory. The total score varies from 0 to 110.

Inhibitory control was assessed by means of *Inhibition* subtest of NEPSY-II battery (Korkman et al., 2007). This subtest is designed to assess the child’s ability to inhibit automatic cognitive responses. The total score has a range of scores from 0 to 19.

#### Play behavior

A caregiver survey was elaborated to assess children’s play behavior. It consisted of 13 statements to be evaluated using a four-point scale. The caregivers were asked to assess how much their child’s behavior matched each statement, assigning it from 1 (this behavior does not take place) to 4 points (this behavior takes place most of the time to always) (see Table 1).

**Table 1:** Correlations and descriptive statistics for all study variables.

	Screen time tantrums	*M*	SD	Min	Max
Child age (months)	−0.018	70.25	4.01	59	82
Caregivers’s education (years)	−0.003	15.16	1.81	9	19
Number of siblings	0.055	0.99	0.78	0	5
Cognitive flexibility	0.024	20.27	2.77	10	24
Working memory	0.013	70.71	19.94	28	120
Inhibitory control	−0.017	10.85	3.15	2	19
Family activities (mean score)	−0.104*	3.75	1.21	1.00	7.00
Play behavior (mean score)	−0.127**	3.02	0.37	1.75	4.00
My child loses interest in new toys literally the same day	0.145***	1.81	0.81	1	4
My child prefers to play with a certain type of toys	0.128**	1.96	0.93	1	4
My child’s favorite toys change depending on current trends	0.125**	2.27	0.94	1	4
My child is emotionally attached to his/her toys	−0.076	2.82	0.87	1	4
My child is not keen to play at all or prefers other activities	−0.041	1.69	0.76	1	4
My child has a designated place to play at home	−0.024	3.02	1.01	1	4
My child gets really involved in the play process	0.016	2.75	0.88	1	4
My child regularly plays with other children	−0.039	3.40	0.75	1	4
My child cannot play alone even for 10 min	0.152***	1.44	0.74	1	4
My child brings his/her toys to play in the kindergarten	−0.001	3.15	0.92	1	4
My child pretends an object is something else in his/her play	0.045	2.66	1.01	1	4
My child likes to play with realistic toys	−0.077	2.63	0.88	1	4
I enjoy participating in my child’s play	−0.129**	2.58	0.76	1	4

(1) Activities mean score corresponded to the average frequency of three types of family activities (reading, singing, and drawing); (2) Play behavior mean score was calculated as an average score of 12 child’s play caregiver-reported characteristics; (3) *p < 0.05, **p < 0.01, ***p < 0.001.

#### Family activities

Family activities were assessed through 3 questions included in the caregiver survey. The caregivers were asked how many times a week they read, drew, and sang together with their child. The responses to choose from, were the following: once a month or less, a couple of times a month, a couple of times a week, several times a week, almost daily, once a day, several times a day.

#### Screen time tantrums

Screen time tantrums were assessed using a question included in the caregiver survey: “Does your child experience painful screen time transitions? “. Caregivers were asked to choose one of the options: no, usually my child does not experience painful screen time transitions; yes, usually my child experiences moderately painful screen time transition (gets upset); yes, my child experiences a highly painful screen time transition (crying, intense negative emotions).

#### Data analysis

Data analysis consisted of two stages. On the first stage, descriptive statistics and preliminary analysis was conducted to explore the data structure and investigate the associations between screen time tantrums EF, play behavior, and family activities. On the second stage, Structural Equation Modeling (SEM) was used to explore preventors of screen time tantrums. The recommendations developed by Byrne (1994) were applied for the evaluation of the SEM Fit Indices: CFI > 0.90; TLI > 0.90; RMSEA < 0.05; SRMR < 0.08.

## Results

### Descriptive statistics and preliminary analysis

The means, standard deviations, and correlations are presented in Table 1. The obtained data indicates that 37.1% of children did not experience screen time tanturms; 55.4% experienced a moderately screen time tanturms; and 7.5% of children experienced a highly painful screen time tanturms. Pearson correlation analysis revealed that the screen time tantrums did not significantly correlate nor with the caregivers’ education, neither the number of siblings, or with the children’s age or main EF skills. Screen time tantrums negatively correlated to play behavior. It is emphasized by the evidence that the children who quickly lost their interest in new toys, preferred to play with a certain type of toys, preferred current popular toys, could not play alone even for 10 min, and whose caregivers did not enjoy being involved in the child’s play, demonstrated more intense screen time tantrums (*p* < 0.05). The frequency of family activities also negatively correlated with the screen time tantrums (the less time caregivers spent reading, singing, and drawing with their children, the more painfully children reacted to screen time limits).

**Figure 1:**
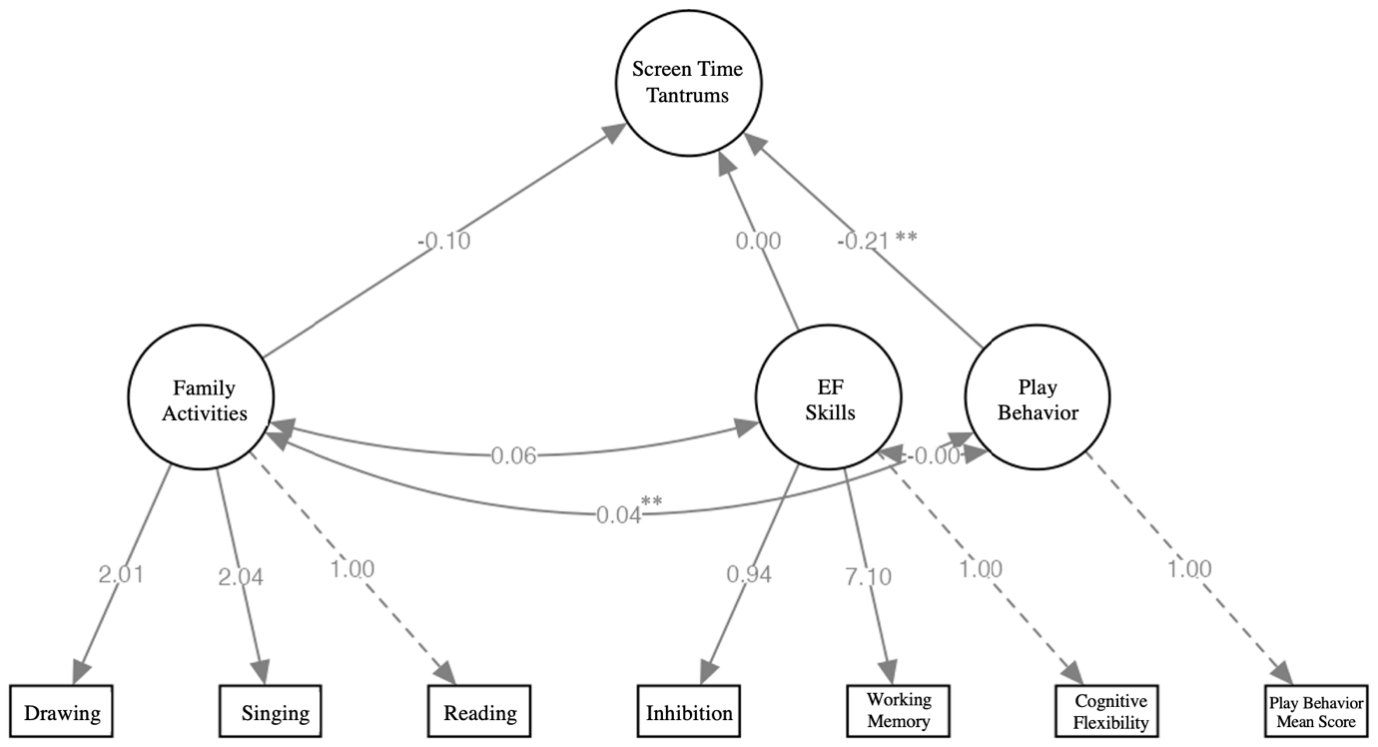
A path diagram in Structural Equation Modeling (SEM); Effects of Play Behavior, EF Skills and Family Activities on Screen Time Tantrums among preschoolers. All variables except EF skills in the presented model were self-reported by caregivers. Standardized coefficients are presented. *p < 0.05, **p < 0.01, ***p < 0.001.

SEM analyses was conducted to test the hypothesis that play behavior was a stronger predictor of screen time tantrum than EF skills (Figure 1). The model fit was adequate, χ^2^(17) = 28.2, *p* = 0.030, CFI = 0.941, TLI = 0.897, RMSEA = 0.038, 90% CI [0.012–0.061], SRMR = 0.031. A direct effect of play behavior on screen time tantrum was registered (*b* = −0.123, SE = 0.077, *p* = 0.007). Neither family activities (*p* = 0.147), nor EF (*p* = 0.964) variables had a significant effect on screen time tantrum in the study participants.

Significant path coefficient *Play Behavior × Screen Time Tantrums* in the diagram indicates that if play behavior increases by one standard deviation from its mean, screen time tantrum expression would be expected to decrease by 0.21 of its own standard deviations from its own mean while holding all other relevant connections constant. The other significant path coefficient *Play Behavior × Family Activities* evidences a bidirectional relationship between play behavior and the frequency of reading, singing, and drawing of caregivers and children together (*b* = 0.234, SE = 0.013, *p* = 0.001).

## Discussion

In the situation of increasing digitalization, it is almost impossible to create a technology-free environment for children (Lemish et al., 2018). Moreover, the use of media is often more attractive for children, than conventional activities. Therefore, frequently, the caregivers’ attempts to enforce screen time limits and occupy their children with alternative non-digital activities causes frustration, resistance, and anger in the latter which results into hysterical behavior called screen time tantrums. Guided by prior research (Hiniker et al., 2016; Coyne et al., 2021; Ishtiaq et al., 2021) and the fundamental theories of play in child development, this study examined two hypotheses. First, whether EF skills, play behavior, and family activities predicted painful screen time tantrums, and second, whether playing with real toys prevented screen time tantrums more efficiently compared to the EF skills. The data, obtained in a sample of children with experience of using media, confirmed that poor play behavior with real toys played a key role in causing screen time tantrums. Thus, play, as a leading activity in preschool age, should be seen as a key to children’s development and learning, but also as a way of setting screen time limits (Smirnova et al., 2019). To our knowledge, this is the first study aimed at the exploring the predictors of screen time tantrums in children.

Our findings make three novel contributions to the scientific understanding of how children experience screen time transition and cope with the associated frustration. Firstly, the attained results indicate that well-developed EF skills in children cannot be considered as an important preventer of screen time tantrums. Secondly, children with more versatile play behavior with real toys demonstrated less screen time tantrums. And third, screen time tantrums are not related to the frequency of such joint family activities as reading, singing, and drawing.

### Play as screen time tantrum preventer

The study results revealed that playing with real toys prevented screen time tantrum among children, while nor EF skills, neither family activities did not cause any significant effect on the painfulness of screen time transitions. This outcome supports the theoretical assumption that play should have a universal ability to reduce emotional tension. This idea was also expressed in such fundamental approaches to child development as psychoanalysis (Wälder, 1933) and the cultural-historical approach (Vygotsky, 1997). However, to the extent of our knowledge, this theory was not supported by empirical evidence.

The fact that children demonstrating advanced play are less prone to painful screen time transitions might be explained by the engrossing alternative to media which is available to them. Yet, the obtained results indicate that joint family activities that could be considered such an alternative did not, in fact, reduce the probability of screen time tantrums. This, in turn, provides the grounds to assume that play has certain characteristics other activities, such as reading, singing, or drawing together with the caregivers, are deprived of. From the perspective of the cultural-historical approach, it is the experience of symbolic fulfillment of the child’s desires that can explain the positive contribution of play to the painless reaction to screen time limits enforcement. Vygotsky pointed out that a child can postpone the fulfillment of his/her desire for a short period of time: “… no one has ever known a child under three who would want to do something in a couple of days. […] I think, if in preschool age, there was no maturation of desires that are impossible to fulfill immediately, there would be no play” (Vygotsky, 1967). The experience of removal of emotional stress in play can help the child to react to screen time transitions and the frustration caused by them, more tranquilly (Veraksa and Sukhikh, 2021). From the psychoanalytical perspective, the role of play in the context of screen time transitions is rather seen as the opportunity to express negative emotions and conflicts symbolically (Wälder, 1933).

### Strengths, limitations, and future directions

The current study adds to research on the new aspects of childhood digitalization, in particular, children’s negative reactions to the screen time limits enforcement. The strengths of this study include the novelty of the research direction, an original theoretical approach, and a large sample. Still, there are limitations. First, all study variables except the EF skills were assessed based on the caregivers’ reports. We addressed this issue by guaranteeing data confidentiality and building non-judgmental statements to avoid social desirability bias. Second, only cold EF skills were measured, as we lacked an efficient tool for an objective assessment of hot EF skills that would be functional on all children and respect gender and individual differences. Future research should clarify the concept of screen time tantrums and develop clear criteria for its assessment, including frequency, duration, intensity, and other parameters. Other important factors that may influence children’s reactions to screen time limits enforcement (caregivers’ screen time, quality of the home environment, child’s temperament, etc.) should also be studied.

## Conclusion

Despite there are a number of studies on the reactions of children and adolescents to screen time limits enforcement, not much is known about the factors that can prevent such reactions in earlier years. The current study fills this gap by demonstrating that play with real toys can positively impact children’s screen time tantrums (i.e., crying, intense negative emotions). The findings support the theoretical assumptions that play is a universal way of coping with frustration and anger in childhood.

Future work in this area should be systematized through meta-analysis to provide a scientific approach to preserving children’s play as a valuable source of development in early childhood and preventing children’s uncontrolled use of media. Nevertheless, the results of this pioneering research can provide preliminary guidance to the parent and teacher community regarding priorities in child development. The findings may also be useful in the development of educational programs for preschool children (arguing in support of children’s play).

## Data availability statement

The raw data supporting the conclusions of this article will be made available by the authors, without undue reservation.

## Ethics statement

The studies involving humans were approved by Ethics Committee of Faculty of Psychology at Lomonosov Moscow State University. The studies were conducted in accordance with the local legislation and institutional requirements. Written informed consent for participation in this study was provided by the participants’ legal guardians/next of kin.

## Author contributions

MG: Data curation, Formal analysis, Investigation, Project administration, Writing – original draft, Writing – review & editing. NV: Conceptualization, Methodology, Supervision, Writing – original draft, Writing – review & editing.
